# Postnatal Changes of Renin and Aldosterone in Term and Preterm Infants from Birth to Day 5

**DOI:** 10.3390/biomedicines14010064

**Published:** 2025-12-27

**Authors:** Yukihito Imagawa, Yu Masuda, Yuki Nakata, Kentaro Fujitani, Aine Takahashi, Keisuke Shirai, Takumi Kido, Mariko Ashina, Kenji Tanimura, Kandai Nozu, Kazumichi Fujioka

**Affiliations:** 1 Department of Pediatrics, Kobe University Graduate School of Medicine, Kobe 650-0017, Hyogo, Japan; yuki0205@med.kobe-u.ac.jp (Y.I.); yumasuda@med.kobe-u.ac.jp (Y.M.); yuki111@med.kobe-u.ac.jp (Y.N.); ken9710@med.kobe-u.ac.jp (K.F.); aine@med.kobe-u.ac.jp (A.T.); ksk1024@med.kobe-u.ac.jp (K.S.); tkido@med.kobe-u.ac.jp (T.K.); marikoa@med.kobe-u.ac.jp (M.A.); nozu@med.kobe-u.ac.jp (K.N.); 2 Department of Obstetrics and Gynecology, Kobe University Graduate School of Medicine, Kobe 650-0017, Hyogo, Japan; taniken@med.kobe-u.ac.jp

**Keywords:** renin, aldosterone, neonates, preterm infants, renin–angiotensin–aldosterone system (RAAS)

## Abstract

**Background/Objectives**: The renin–angiotensin–aldosterone system (RAAS) is pivotal for neonatal circulation and renal adaptation; however, postnatal changes in serum renin and aldosterone immediately after birth remain unclear. This study aimed to establish postnatal changes in these hormones at birth and over the first week of life. **Methods**: We retrospectively analyzed 374 neonates admitted to Kobe University Hospital between October 2020 and September 2023, with serum renin and aldosterone measured on days 0 and 5 of life. Exclusion criteria were multiple congenital anomalies, severe asphyxia, major peripartum hemorrhage, and in utero exposure to angiotensin-converting enzyme inhibitors or angiotensin II receptor blockers. Hormone levels were compared between term and preterm infants, and correlations with gestational age were assessed. **Results**: Serum renin concentrations were higher on day 0 than on day 5 (median 99.9 pg/mL [2.6–773.3] vs. 19.9 pg/mL [0.6–2304], *p* < 0.0001), and aldosterone concentrations similarly decreased (714 pg/mL [6.9–6334] vs. 551 pg/mL [0–11,930], *p* < 0.0001). At birth, renin and aldosterone levels did not differ significantly between groups. By day 5, both renin (32.8 pg/mL [0.6–2304] vs. 14.5 pg/mL [0.6–208]) and aldosterone (689 pg/mL [4–11,930] vs. 471 pg/mL [13–4697]) concentrations were significantly higher in preterm than in term neonates (*p* < 0.0001). **Conclusions**: This study describes early postnatal changes in renin and aldosterone, with higher concentrations at birth than on day 5 and persistently elevated levels in preterm infants. These findings indicate increased RAAS activity in preterm neonates and suggest a greater vulnerability to fluid, electrolyte, and blood pressure instability during early life.

## 1. Introduction

The renin–angiotensin–aldosterone system (RAAS) is a major hormonal pathway that regulates blood pressure and fluid homeostasis. The juxtaglomerular apparatus of the kidney secretes renin in response to decreased renal perfusion pressure, reduced sodium or chloride ion concentrations in the distal tubular filtrate, or stimulation of β1-adrenergic receptors. Renin indirectly stimulates aldosterone secretion through activation of the renin–angiotensin cascade. Aldosterone acts on the distal tubules and collecting ducts of the kidney to enhance sodium reabsorption and potassium excretion, thereby increasing intravascular volume and arterial pressure [1]. In neonates, the RAAS responds dynamically to rapid postnatal fluctuations in intravascular volume and electrolyte concentrations [2]. Elevated plasma renin activity and serum aldosterone concentrations have also been observed in a subset of preterm infants with hypertension [3]. RAAS components are expressed as early as the fifth week of gestation in the human fetus, and their production is tightly regulated in a temporally specific pattern [4]. Furthermore, studies by Barr and Pryde have reported that fetal exposure to angiotensin-converting enzyme (ACE) inhibitors or angiotensin II type 1 (AT1) receptor blockers can cause severe neonatal hypotension and irreversible renal injury, often leading to renal failure or anuria [5,6]. These findings indicate that the RAAS plays a critical role in fetal renal development and maturation [7]. Collectively, these findings underscore the essential role of the RAAS is a crucial hormonal system both before and after birth. 

Renin and aldosterone have also been implicated in the pathogenesis of pulmonary [8], cardiovascular [9], and renal disorders [10] in preterm infants, suggesting a pivotal role of the RAAS in the pathophysiology of prematurity. In the early neonatal period, reduced mineralocorticoid receptor expression results in physiological partial aldosterone resistance [11]. Consequently, neonates exhibit higher renin and aldosterone concentrations than infants and adults, yet paradoxically demonstrate increased urinary sodium excretion. Stephenson et al. reported that the renin concentrations measured during the first postnatal week in 52 infants born before 37 weeks’ gestation were significantly higher than those in adults [12]. In a more recent study in 2005, Bourchier et al. reported a similar inverse relationship between aldosterone levels and gestational age in 50 preterm infants born before 30 weeks of gestation [13].

Furthermore, Xu et al. observed a negative correlation between renin and aldosterone concentrations at birth and gestational age in 262 hemodynamically stable preterm infants born between 26 and 34 weeks’ gestation [14]. These findings indicate that the RAAS is upregulated in preterm infants compared with term infants; however, due to renal tubular unresponsiveness, sodium excretion remains elevated. Nevertheless, data on postnatal changes in the RAAS during the early neonatal period, including in preterm infants, remain limited. Several studies have investigated the postnatal trajectory of renin and aldosterone concentrations in neonates. A pioneering 1972 study involving 20 neonates demonstrated for the first time that the RAAS, assessed through plasma renin activity (PRA) and renin substrate concentration, was more active in neonates than in adults. PRA and renin substrate concentrations increase from 24 h after birth to approximately 3–6 days, then decline to below early neonatal levels by 3–6 weeks of age [15]. Additionally, Bourchier et al. reported that aldosterone concentrations increased from day 1 to day 7 of life in 50 preterm infants born before 30 weeks’ gestation [13]. Similarly, Xu et al. found that in a cohort of 380 clinically stable preterm infants across various gestational ages, both renin and aldosterone concentrations increased between days 1 and days 14–21, indicating a postnatal increase during the early neonatal period [14].

As noted above, only a few studies have reported postnatal changes in renin and aldosterone concentrations in preterm infants. However, to date, no study has comprehensively investigated the early postnatal trajectory or established reference ranges of these hormones, including measurements at birth, in a large cohort of both preterm and term infants. Therefore, this study aimed to examine the postnatal ranges and temporal changes in renin and aldosterone concentrations during the early neonatal period in a cohort of preterm and term neonates.

## 2. Materials and Methods

We included neonates admitted to the Center for Perinatal Care at Kobe University Hospital between 1 October 2020, and 30 September 2023, who underwent serum renin and aldosterone measurements on days 0 and 5 of life. The exclusion criteria were as follows: in utero exposure to drugs known to cause congenital malformations or nephrotoxicity (e.g., ACE inhibitors and AT1 receptor blockers), presence of congenital anomalies or chromosomal abnormalities; severe perinatal asphyxia, defined as umbilical cord blood pH < 7.0 or a 5 min Apgar score < 3, and delivery complicated by massive peripartum hemorrhage (e.g., due to placental abruption or placenta previa), based on previous literature [14].

Maternal data were extracted from electronic medical records and included maternal age at delivery, height, pre-pregnancy weight, parity, multiple pregnancies, mode of delivery, premature rupture of membranes (PROM), presence of diabetes or hypertensive disorders of pregnancy (HDP), use of tocolytic agents (magnesium sulfate and ritodrine hydrochloride), antenatal steroid administration (betamethasone), calcium channel blocker use, and placental pathological abnormalities. Neonatal data included sex, gestational age, birth weight, Apgar scores at 1 and 5 min, small for gestational age (SGA) status, use of inotropic agents (dopamine and dobutamine) and diuretics (furosemide), and serum renin and aldosterone levels on days 0 and 5. Neonatal asphyxia was defined as an Apgar score ≤ 6 at 5 min. SGA was defined as birth weight below the 10th percentile of the mean value for Japanese newborns of the same gestational age [16].

Blood samples were promptly centrifuged, and the resulting serum was stored at −80 °C until analysis. Renin and aldosterone concentrations were measured using 170 μL of serum with a chemiluminescent enzyme immunoassay (CLEIA), employing the Lumipulse Presto Renin Kit and Lumipulse Presto Aldosterone Kit (Fujirebio Inc., Tokyo, Japan) according to the manufacturer’s instructions [17,18].

First, we assessed the distribution of serum renin and aldosterone levels on days 0 and 5 in the entire cohort. Neonates were then categorized into term (≥37 weeks of gestation) and preterm (<37 weeks) groups, and their clinical characteristics and hormone levels were compared. Finally, multivariate analyses were performed to assess whether gestational age independently influenced renin and aldosterone levels after adjusting for maternal and neonatal factors.

Continuous variables are expressed as medians (range) or means ± standard deviation (SD). Paired comparisons were performed using the Wilcoxon signed-rank test, and comparisons between independent groups were conducted using the Mann–Whitney U test. Categorical variables were compared using Fisher’s exact test. Correlations between continuous variables were assessed using Spearman’s rank correlation coefficients. Multivariate linear regression was performed using the standard least-squares method. For the multivariate analysis, variables were selected based on clinical relevance and significant differences between term and preterm infants in univariate comparisons. Factors strongly correlated with gestational age (e.g., birthweight and Apgar scores) were excluded to avoid multicollinearity. Postnatal weight change was analyzed as a continuous variable. Enteral feeding was categorized into four groups (breast milk, mixed feeding, formula feeding, and no enteral feeding), and these variables were incorporated into the multivariate regression models. Statistical significance was set at *p* < 0.01. Statistical analyses were performed using GraphPad Prism version 10 (GraphPad Software, La Jolla, CA, USA) and JMP Pro 17 (SAS Institute, Cary, NC, USA).

## 3. Results

**Figure 1:**
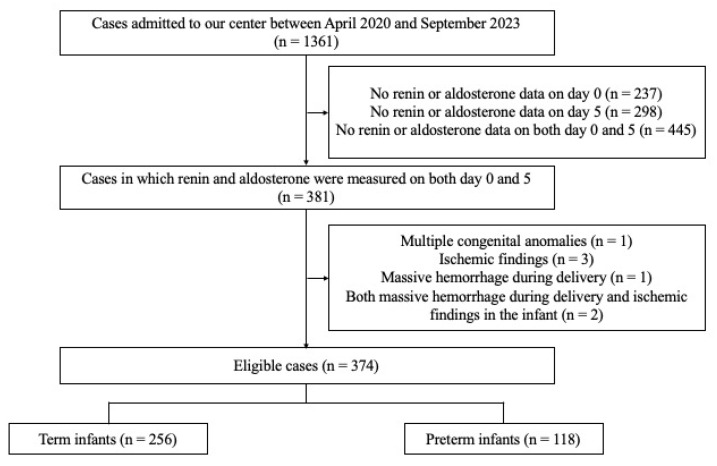
Flow chart of case selection. Among 1361 infants admitted between April 2020 and September 2023, 374 cases with renin and aldosterone measured on both day 0 and day 5 were eligible for analysis (256 term and 118 preterm infants).

Between 1 October 2020, and 30 September 2023, 1361 neonates were admitted to the Center for Perinatal Care at Kobe University Hospital. Among these, 381 neonates underwent serum renin and aldosterone measurements on days 0 and 5, respectively. The exclusion criteria were met by seven neonates: one with multiple congenital anomalies or chromosomal abnormalities, three with severe ischemic findings at birth (defined as an umbilical artery pH < 7.0 or a 5 min Apgar score < 3), one with significant peripartum hemorrhage, and two with both massive peripartum hemorrhage and neonatal ischemic findings. None of the neonates had been exposed in utero to angiotensin-converting enzyme inhibitors or angiotensin II receptor blockers. After applying the exclusion criteria, 374 neonates were included in the final analysis (Figure 1).

### 3.1. Patient Characteristics

The gestational age of the study population ranged from 23 weeks and 5 days to 41 weeks and 4 days, with a median of 37 weeks and 4 days. The median birth weight was 2734 g (range: 562–4195 g), and 179 neonates (47.9%) were male. The median Apgar score was 8 (range: 1–9) at 1 min and 9 (range: 3–10) at 5 min.

With respect to maternal characteristics, the median maternal age was 34 years (range: 18–44), and the median height and weight at pregnancy were 158 cm (range: 144–180.5) and 61 kg (range: 40.4–122.5), respectively. Multiple pregnancies were significantly more frequent in the preterm group (23.7%) than in the term group (3.5%; *p* < 0.001). Cesarean delivery was performed in 68.7% of cases, comprising 42.0% elective and 26.7% emergency procedures. Emergency cesarean delivery was significantly more common in preterm neonates (54.2%) than in term neonates (14.1%; *p* < 0.001). Antenatal steroid administration occurred exclusively in preterm pregnancies (18.6%; *p* < 0.001). Tocolytic agents were used more frequently in preterm pregnancies (52.5%) than in term pregnancies (9.4%; *p* < 0.001), including ritodrine hydrochloride (35.6%), magnesium sulfate (1.7%), and combined therapy (15.3%). Placental abnormalities were present in 73.4% of available samples, with no significant differences between the term and preterm groups. However, placental pathological examination was performed more frequently in preterm infants than in term infants (77.1% vs. 41.4%, *p* < 0.0001).

Among the 256 term infants, 64 were admitted due to maternal factors, including maternal infections (e.g., cytomegalovirus, Toxoplasma gondii, or syphilis), diabetes or gestational diabetes, hypertensive disorders of pregnancy, thyroid disease, and maternal medication use such as antianxiety or psychotropic drugs. These infants were admitted mainly for screening and observation, and none required specific treatment. The remaining 192 term infants were hospitalized for neonatal or perinatal conditions, most commonly transient tachypnea of the newborn (*n* = 153), of whom 101 required temporary endotracheal intubation for respiratory support. Other reasons for admission included neonatal hypoglycemia (*n* = 8), mild birth asphyxia, feeding difficulties, low birth weight, and minor congenital anomalies (e.g., hemangioma and hydronephrosis). Overall, most term infants were admitted for mild and transient perinatal conditions rather than severe pathological disorders.

The median gestational age at birth was 38.3 weeks in the term group and 35.1 weeks in the preterm group (*p* < 0.001). The median birth weight was significantly lower in preterm neonates (2174 g) than in term neonates (2890 g; *p* < 0.001). Apgar scores at both 1 and 5 min were significantly lower in the preterm group than in the term group (both *p* < 0.001). The incidence of neonatal asphyxia did not differ significantly between the groups (1.7% vs. 1.2%). The proportion of infants who initiated enteral feeding within the first 5 days of life was significantly lower in the preterm group than in the term group (66.1% vs. 97.3%, *p* < 0.001). Likewise, the proportion of infants with physiological weight loss (<10%) was significantly lower among preterm infants compared with term infants.

Inotropic agents were administered more frequently in preterm neonates (44.9%) than in term neonates (3.9%, *p* < 0.001), with dopamine used in 8.5% and dopamine plus dobutamine in 36.4% of preterm infants. Diuretic therapy was significantly more common in preterm infants (49.2%) than in term infants (10.6%; *p* < 0.001). The overall incidence of small-for-gestational-age neonates was 7.8%, with no significant difference between the two groups (Table 1).

### 3.2. Renin, Aldosterone, and Aldosterone-to-Renin Ratio at Birth

Serum renin concentrations were significantly higher on day 0 than on day 5, with a median of 99.9 pg/mL (range: 2.6–773.3) on day 0 and 20.0 pg/mL (range: 0.6–2304) on day 5 (*p* < 0.0001). Similarly, aldosterone concentrations were significantly higher on day 0 than on day 5, with a median of 714.2 pg/mL (range: 6.9–6334.1) on day 0 and 551.0 pg/mL (range: 0–11,930.3) on day 5 (*p* < 0.0001) The aldosterone-to-renin ratio was lower on day 0 (median 7.4 [0.1–95.2]) than on day 5 (median 28.8 [0.0–989.5]) (Table 2).

**Figure 2:**
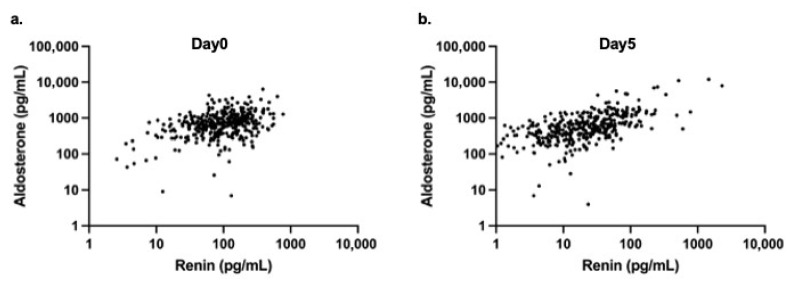
Scatter plots showing the relationship between serum renin and aldosterone concentrations in neonates. (**a**) Day 0 (at birth) and (**b**) Day 5 of life. Both renin and aldosterone levels are presented on logarithmic scales.

A moderate positive correlation was observed between renin and aldosterone levels on day 0 (r = 0.33, *p* < 0.0001) (Figure 2a), and a stronger positive correlation was identified on day 5 (r = 0.65, *p* < 0.0001) (Figure 2b).

### 3.3. Comparison of Renin and Aldosterone Levels Between Preterm and Term Neonates

Among the study population, 256 neonates were classified as term (median gestational age: 38 weeks 2 days; range: 37 weeks 0 days to 41 weeks 4 days) and 118 as preterm (median gestational age: 35 weeks 1 day; range: 23 weeks 5 days to 36 weeks 6 days). The median birth weight was 2890 g (range: 1826–4195 g) in the term group and 2174 g (range: 562–3386 g) in the preterm group (Table 1). Significant differences in clinical characteristics were observed between term and preterm neonates, including birth weight, incidence of multiple pregnancies, Apgar scores at 1 and 5 min, and mode of delivery, with vaginal delivery occurring more frequently in the term group.

On day 0, serum renin levels did not differ significantly between term neonates (median: 104.2 pg/mL; range: 9.9–557.1) and preterm neonates (median: 92.3 pg/mL; range: 2.6–773.3) (*p* = 0.2791). Similarly, aldosterone levels on day 0 were comparable between term and preterm groups (term: 738.6 pg/mL [range: 6.9–6334.0]; preterm: 682.7 pg/mL [range: 9.0–4263.0]; *p* = 0.06) (Table 2). In contrast, on day 5, both renin and aldosterone levels were significantly higher in preterm neonates. Renin levels were 32.8 pg/mL (range: 0.6–2304.0) in preterm neonates and 14.5 pg/mL (range: 0.6–207.5) in term neonates (*p* < 0.0001). Aldosterone levels were also elevated in the preterm group (688.8 pg/mL; range: 0–11,930.0) than in the term group (470.8 pg/mL; range: 13.0–4697.0) (*p* < 0.0001) (Table 2).

**Figure 3:**
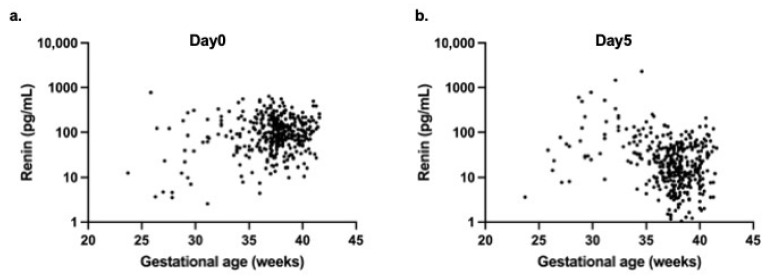
Scatter plots showing the relationship between gestational age at birth and serum renin concentrations in neonates. (**a**) Day 0 (at birth) and (**b**) Day 5 of life. Renin concentrations are presented on a logarithmic scale.

Correlation analyses revealed no significant association between gestational age and renin levels on day 0 (r = 0.083, *p* = 0.11) (Figure 3a). However, a significant negative correlation was observed on day 5 (r = −0.242, *p* < 0.0001) (Figure 3b).

**Figure 4:**
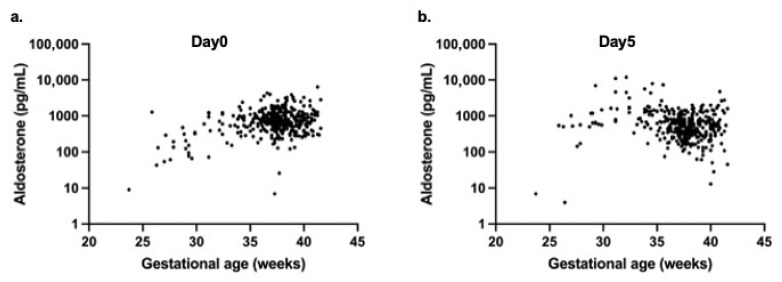
Scatter plots showing the relationship between gestational age at birth and serum aldosterone concentrations in neonates. (**a**) Day 0 (at birth) and (**b**) Day 5 of life. Aldosterone concentrations are presented on a logarithmic scale.

For aldosterone, a weak positive correlation with gestational age was noted on day 0 (r = 0.143, *p* = 0.01) (Figure 4a), whereas a significant negative correlation was observed on day 5 (r = −0.198, *p* < 0.0001) (Figure 4b).

### 3.4. Multivariate Analyses to Assess the Independent Impact of Gestational Age

Multivariate linear regression was performed to evaluate whether gestational age independently influenced renin and aldosterone levels after adjusting for maternal and neonatal factors. For Day 0 renin and aldosterone, explanatory variables included gestational age, multiple pregnancies, cesarean section, use of antenatal steroids, use of tocolytic agents, and SGA status. For Day 5 renin and aldosterone levels, explanatory variables included gestational age, multiple pregnancies, cesarean section, use of antenatal steroids, use of tocolytic agents, use of inotropic agents, use of diuretics, and SGA status. The values were log-transformed prior to the analysis because the distributions of renin and aldosterone were skewed.

In multivariate regression analysis at day 0, gestational age was positively associated with log-transformed aldosterone levels (β = 0.09, *p* < 0.0001), but not with renin levels. Multiple pregnancy was significantly associated with lower aldosterone levels (β = −0.19, *p* < 0.01). The use of antenatal steroids was associated with higher levels of renin (β = 0.46, *p* < 0.001) and aldosterone (β = 0.35, *p* < 0.001). The other variables showed no significant associations (Table 3).

In multivariate regression analysis on day 5, gestational age was not significantly associated with log-transformed renin or aldosterone levels. The use of diuretics was the only variable significantly associated with aldosterone levels (β = −0.23, *p* < 0.001). All other variables showed no significant associations (Table 4).

Additional multivariable analyses for the aldosterone-to-renin ratio on days 0 and 5 were conducted, and the results are presented in Supplementary Tables S1 and S2.

## 4. Discussion

In this study, we present, for the first time, postnatal change for serum renin and aldosterone concentrations at birth (day 0) in a large neonatal cohort and demonstrate a significant decline in both hormone levels by day 5 of life. Additionally, although no significant differences in serum renin and aldosterone concentrations were observed between preterm and term infants at birth (day 0), both hormone levels were significantly higher in preterm infants than in term infants by day 5 of life.

We measured serum renin and aldosterone concentrations on days 0 and 5 in 374 neonates and confirmed a significant decrease during the early postnatal period (*p* < 0.0001). Kotchen et al. assessed renin levels within the first 12 h of life in 20 healthy neonates and reported levels higher than those observed in adults [19]. They also observed an increase in renin concentrations between 24 h and 3–6 days after birth, followed by a decline at 3–6 weeks compared with the early neonatal period. However, the reliability of their findings was limited by the small sample size and the exclusive inclusion of healthy neonates. Similarly, Bourchier et al. measured aldosterone concentrations during the first week of life in 50 preterm infants born at less than 30 weeks of gestation and reported an increase from day 1 to day 7 [13]. Nonetheless, this study is constrained by its exclusive focus on preterm infants, the absence of immediate postnatal measurements (starting instead at 24 h of age), and a smaller sample size than ours, which limited the reliability of its conclusions. Furthermore, Xu et al. evaluated renin and aldosterone concentrations in 380 clinically stable preterm infants born at 26–34 weeks of gestation, using measurements obtained between 6 h after birth and 14–21 days of age [14]. They reported increased levels of both hormones after birth. However, this study is also limited by its exclusive focus on preterm infants and by performing the second set of measurements after two weeks of age, which prevented adequate assessment of early neonatal changes. Collectively, these previous studies were limited by their exclusive focus on either preterm or term infants, without enabling a direct comparison between the two within the same cohort. Moreover, none of these studies evaluated renin and aldosterone levels immediately after birth, before therapeutic intervention, which is a critical gap in understanding early neonatal physiology. Our study uniquely analyzed a large cohort of 374 infants, including both preterm and term neonates, allowing direct comparison between the groups. Importantly, we measured renin and aldosterone concentrations immediately after birth and demonstrated for the first time that these levels were higher at birth than during the first week of life. This finding offers novel insights that differ from previous studies. Two mechanisms may underlie the decline by day 5: (i) resolution of acute perinatal RAAS activation during circulatory transition and (ii) early postnatal changes in volume and sodium balance, together with renal maturation, leading to RAAS downregulation. In addition, part of the observed decline from day 0 to day5 may reflect postnatal therapeutic and nutritional influences. In multivariate analyses, diuretic use was significantly associated with lower aldosterone levels, and the timing of enteral feeding initiation and physiological weight loss were also significant determinants of hormone concentrations. These findings suggest that treatment- and nutrition-related factors, in addition to developmental physiology, contribute to the hormonal changes observed on day 5. To further explore factors influencing hormone levels at birth, we examined the relationship between day-0 aldosterone concentrations and birthweight (Supplementary Figure S1). A distributional pattern similar to that observed with gestational age was noted (r = 0.145, *p* < 0.01), suggesting that both overall body size and maturational factors may contribute to the variability in aldosterone levels at birth.

We also found no significant differences in hormone levels at birth between term and preterm infants. Serum renin and aldosterone concentrations were significantly higher in preterm infants and showed a negative correlation with gestational age by day 5 of life. In multivariate regression analyses, however, gestational age was independently associated with aldosterone levels at birth, but not with renin or day 5 hormone levels. Maternal steroid administration strongly influenced day 0 hormone levels. On day 5, diuretic use was the only postnatal factor independently associated with aldosterone concentrations, whereas no other variables showed significant associations. Stephenson evaluated 52 preterm infants born at less than 37 weeks of gestation and found renin concentrations during the first week of life to be significantly higher than those in adults [12]. Consistent with this report, we found that renin levels in preterm infants were higher than adult reference values on days 0 and 5. Bourchier et al. evaluated 50 preterm infants born at less than 30 weeks’ gestation and reported an inverse correlation between plasma aldosterone concentrations during the first week of life and gestational age [13]. Xu et al. found no statistically significant correlations between gestational age and either renin or aldosterone concentrations [14]. However, premature infants tended to exhibit higher renin levels at 14–21 days of age and lower aldosterone levels on day 1, suggesting a partial association with gestational age. In contrast, although no significant differences were observed in hormone concentrations at birth between term and preterm infants, by day 5, both renin and aldosterone levels were significantly higher in preterm infants and negatively correlated with gestational age. These findings suggest that preterm infants may remain in a state of increased RAAS activity after birth, partially aligning with the observations of Xu et al. [14].

These findings have important clinical implications for managing neonates during the early postnatal period. Renin and aldosterone levels peak at birth and decline during the first week of life, underscoring the dynamic role of the RAAS in circulatory adaptation. The persistence of higher hormone levels in preterm infants suggests increased susceptibility to fluid and electrolyte disturbances, blood pressure instability, and potential long-term cardiovascular or renal outcomes. These observations should be interpreted in the context of the well-documented relative aldosterone resistance in neonatal renal tubules [11,20], particularly in preterm infants [21]. Despite elevated circulating aldosterone concentrations, neonates often exhibit sodium wasting, hyperkalemia, and impaired fluid balance due to the immature tubular responsiveness. Moreover, RAAS activation may also interact with developmental signaling pathways such as TGF-β, which has been shown to influence cellular maturation and autophagy-related processes [22]. Thus, the elevated renin and aldosterone levels observed in preterm infants in this study may reflect compensatory RAAS activation in response to physiological aldosterone resistance, as well as ongoing hemodynamic adaptation and renal immaturity during early infancy.

This study has several limitations. First, as a retrospective analysis, the study was subject to inherent biases in data collection and patient selection. Second, although the cohort was relatively large compared with previous reports, the sample size remained insufficient to define reference values across the full spectrum of gestational ages, particularly because the number of extremely immature infants (e.g., ELBW and VLBW) was limited. Moreover, the single-ethnicity, single-center cohort limits the broader applicability of the results. Third, serum renin and aldosterone concentrations were measured at only two time points (days 0 and 5), which may not have fully captured the dynamic changes in RAAS activity during the early postnatal period. In addition, because day 0 samples were obtained before any clinical intervention whereas day 5 samples were collected after several days of postnatal management, these measurements did not constitute a continuous longitudinal trajectory. For this reason, advanced longitudinal models such as generalized estimating equations were not applied, as their assumptions regarding within-subject dependence were not met in this dataset. Fourth, particularly in preterm infants on day 5, the influence of postnatal circulatory management and other clinical interventions on hormone levels could not be excluded, a limitation shared by previous studies in this field. Future prospective multicenter studies with larger cohorts and serial measurements are required to validate and expand these findings. Finally, although this study provided valuable insights into postnatal RAAS activity, it was not designed to establish definitive reference ranges for renin and aldosterone in neonates. To define true “normal” or “reference” ranges, only strictly healthy reference individuals should be included, using validated and standardized assays with documented analytical performance, and statistical procedures should confirm data normality and exclude outliers according to accepted guidelines (e.g., CLSI EP28-A3c). Future well-designed prospective studies adhering to these standards are required to establish robust reference intervals in this population.

## 5. Conclusions

In conclusion, this study demonstrated that serum renin and aldosterone concentrations were higher at birth than on day 5 of life, indication early postnatal changes in RAAS activity. Although hormone levels at birth did not differ between term and preterm infants, both renin and aldosterone remained significantly higher in preterm infants by day 5. Multivariate analyses further identified several perinatal factors—including multiple pregnancy, the use of antenatal steroids, and diuretic therapy—that independently influenced postnatal RAAS activity.

These findings highlight the dynamic nature of the RAAS during the early neonatal period and underscore the particular vulnerability of preterm infants to disturbances in fluid balance, electrolyte homeostasis, and blood pressure regulation. Clinically, the results indicate that interpretation of renin and aldosterone levels in neonates requires careful consideration of feeding status, weight change, and postnatal therapeutic interventions, and they suggest that RAAS biomarkers may contribute to individualized circulatory and renal management in the NICU.

From a broader public health perspective, improved understanding of postnatal RAAS physiology may aid in the development of strategies to optimize early cardiovascular and renal adaptation, particularly in high-risk preterm infants. Establishing reliable reference values across gestational ages could also support early identification of infants at risk for long-term cardiorenal complications.

Future prospective multicenter studies with larger and more diverse cohorts—including extremely and very preterm infants, multiple standardized sampling points, and multiethnic, population-based populations—are warranted to validate these findings and to develop robust, generalizable gestation-specific reference intervals for renin and aldosterone.

## Figures and Tables

**Table 1:** Patient characteristics.

	Overall (*n* = 374)	Term (*n* = 256)	Preterm (*n* = 118)	*p*-Value
Maternal				
Age at pregnancy (years)	34 [18, 44]	34 [18, 44]	33 [23, 44]	0.08
Height (cm) (*n* = 365)	158 [144, 180.5]	158 [146, 180.5]	159 [144, 173]	0.03
Weight (kg) (*n* = 339)	61 [40.4, 122.5]	61.3 [40.4, 122.5]	60.1 [44.2, 96.2]	0.05
Primiparity (%)	186 (49.7)	130 (50.8)	56 (47.5)	0.58
Multiple pregnancy (%)	37 (9.7)	9 (3.5)	28 (23.7)	<0.001
Cesarean section (%)	257 (68.7)	154 (60.2)	103 (87.3)	<0.001
Elective (%)	157 (42.0)	118 (46.1)	39 (33.1)	<0.01
Emergency (%)	100 (26.7)	36 (14.1)	64 (54.2)	<0.001
Premature rupture of membranes, PROM (%)	45 (12.0)	27 (10.5)	18 (15.3)	0.23
Hypertensive disorders of pregnancy (%)	26 (7.0)	19 (7.4)	7 (5.9)	0.67
Gestational diabetes (%)	52 (14.0)	37 (14.5)	15 (12.7)	0.75
Maternal diabetes (%)	8 (2.1)	5 (2.0)	3 (2.5)	0.71
Use of antenatal steroids (%)	22 (6.7)	0 (0)	22 (18.6)	<0.001
Use of calcium channel blocker (%)	14 (3.2)	9 (3.5)	5 (4.2)	0.77
Use of tocolytic agents (%)	86 (23.0)	24 (9.4)	62 (52.5)	<0.001
Ritodrine hydrochloride only (%)	62 (16.6)	20 (7.8)	42 (35.6)	<0.001
Magnesium sulfate only (%)	5 (1.3)	3 (1.2)	2 (1.7)	0.65
Both Ritodrine + Magnesium (%)	19 (5.1)	1 (0.4)	18 (15.3)	<0.001
Placental pathological examination performed	197 (52.7)	106 (41.4)	91 (77.1)	<0.001
Any abnormal findings ^1^	145 (73.4)	79 (74.5)	66 (72.5)	0.87
Findings suggestive of HDP (small infarcts or infarctions) ^1^	16 (8.1)	9 (8.5)	7 (7.7)	>0.99
Villous necrosis/infarction ^1^	3 (1.5)	1 (0.9)	2 (2.2)	0.60
Chorioamnionitis (CAM) ^1^	63 (32.0)	41 (38.7)	22 (24.2)	0.03
Fibrin deposition ^1^	40 (20.3)	24 (22.6)	16 (17.6)	0.48
Microcalcification/small calcification ^1^	12 (6.1)	3 (2.8)	9 (9.9)	0.07
Chorangiosis ^1^	8 (4.1)	1 (0.9)	7 (7.7)	0.03
Other minor findings ^1,2^	3 (1.5)	0 (0)	3 (3.3)	0.10
Neonatal				
Gestational age (weeks)	37.6 [23.7, 41.6]	38.3 [37.0, 41.6]	35.1 [23.7, 36.9]	<0.001
Birth weight (g)	2734 [562, 4195]	2890 [1826, 4195]	2174 [562, 3386]	<0.001
Male (%)	179 (47.9)	123 (48)	56 (47)	1.00
Apgar score at 1 min	8 [1, 9]	8 [1, 9]	8 [1, 9]	<0.001
Apgar score at 5 min	9 [3, 10]	9 [3, 10]	9 [4, 9]	<0.001
Neonatal Asphyxia (%)	5 (1.3)	3 (1.2)	2 (1.7)	0.65
Small for gestational age, SGA	29 (7.8)	17 (6.6)	12 (10)	0.30
Use of inotropic agents (%)	63 (16.8)	10 (3.9)	53 (44.9)	<0.001
Dopamine (%)	12 (3.2)	2 (0.8)	10 (8.5)	<0.001
Dopamine + Dobutamine (%)	51 (13.6)	8 (3.1)	43 (36.4)	<0.001
Use of diuretics (%)	85 (22.7)	27 (10.6)	58 (49.2)	<0.001
Initiation of enteral feeding within 5 days (%)	327 (87.4)	249 (97.3)	78 (66.1)	<0.001
Breast feeding (%)	14 (3.7)	5 (2.0)	9 (7.6)	0.04
Mixed feeding (%)	290 (77.5)	226 (88.3)	64 (54.2)	<0.001
Formula feeding (%)	23 (6.1)	18 (7.0)	5 (4.2)	0.62
Body weight measured on day 5 (%)	330 (88.2)	250 (97.7)	80 (67.8)	<0.001
No weight loss (%) ^3^	32 (8.5)	31 (12.1)	1 (0.1)	<0.001
Physiological weight loss <10% (%) ^3^	282 (75.4)	218 (85.2)	64 (54.2)	<0.001
Excessive weight loss ≥10% (%) ^3^	16 (4.8)	1 (0.4)	15 (12.7)	<0.001

Data are presented as the median [range] or number (%). *p*-values indicate comparisons between term and preterm infants. The Mann–Whitney U test was used for comparisons between term and preterm infants. ^1^ Among cases with placental pathological examination. ^2^ Other minor findings include combined CAM and HDP findings (*n* = 1), hemosiderin deposition (*n* = 1), and villous edema (*n* = 1). ^3^ Weight change from birth to day 5 was calculated as (day 5 weight − birth weight)/birth weight × 100.

**Table 2:** Serum renin and aldosterone levels at birth (Day 0) and on Day 5 in preterm and term neonates.

Condition	Group (*n*)	Median [Range]	Mean ± SD	*p*-Value (Preterm vs. Term)	*p*-Value (Day 0 vs. Day 5)
Renin day 0	All (374)	99.9 [2.6, 773.3]	131.8 ± 112.7	—	<0.0001
Renin day 0	Preterm (118)	92.3 [2.6, 773.3]	136.2 ± 138.5	0.28	
Renin day 0	Term (256)	104.2 [9.9, 557.1]	129.7 ± 98.8		
Renin day 5	All (374)	20.0 [0.6, 2304]	50.3 ± 156.3	—	
Renin day 5	Preterm (118)	32.8 [0.6, 2304]	104.2 ± 268.0	<0.0001	
Renin day 5	Term (256)	14.5 [0.6, 207.5]	25.4 ± 28.7		
Aldosterone day 0	All (374)	714.2 [6.9, 6334]	876.2 ± 714.7	—	<0.0001
Aldosterone day 0	Preterm (118)	682.7 [9.0, 4263]	789.5 ± 688.4	0.06	
Aldosterone day 0	Term (256)	738.6 [6.9, 6334]	916.2 ± 724.3		
Aldosterone day 5	All (374)	551.0 [4, 11, 930]	827.9 ± 1196	—	
Aldosterone day 5	Preterm (118)	688.8 [4, 11, 930]	1289 ± 1909	<0.0001	
Aldosterone day 5	Term (256)	470.8 [13.0, 4697]	615.4 ± 524.2		
Aldosterone-to-renin ratio day 0	All (374)	7.4 [0.1, 95.2]	11.5 ± 12.4		<0.0001
Aldosterone-to-renin ratio day 0	Preterm (118)	7.5 [0.5, 95.2]	12.9 ± 16.1	0.99	
Aldosterone-to-renin ratio day 0	Term (256)	7.4 [0.1, 58.3]	10.8 ± 10.2		
Aldosterone-to-renin ratio day 5	All (374)	28.8 [0.0, 989.5]	53.3 ± 86.9		
Aldosterone-to-renin ratio day 5	Preterm (118)	21.1 [0.0, 233.7]	33.6 ± 40.9	0.0002	
Aldosterone-to-renin ratio day 5	Term (256)	34.4 [2.2, 989.5]	62.3 ± 100.1		

Data are presented as median [range] and mean ± SD. *p*-values indicate comparisons between groups. The Mann–Whitney U test was applied to compare term and preterm infants. The Wilcoxon matched-pairs signed-rank test was applied for within-group comparisons between days 0 and 5.

**Table 3:** Multivariable Linear Regression Analysis of Factors Associated with Log-Transformed Renin and Aldosterone Levels at Day 0.

Variable	Log-Transformed Renin		Log-Transformed Aldosterone	
	β	*p*-Value	β	*p*-Value
Gestational age (weeks)	0.03	0.28	0.09	<0.0001
Multiple pregnancy	0.20	0.05	−0.19	<0.01
Cesarean section	0.04	0.49	−0.03	0.59
Use of antenatal steroids	0.46	<0.001	0.35	<0.001
Use of tocolytic agents	0.02	0.83	−0.09	0.12
SGA	−0.08	0.41	0.03	0.70

Data are multivariable linear regression coefficients (β) with *p*-values. The results were log-transformed. The significance threshold was set at *p* < 0.01.

**Table 4:** Multivariable Linear Regression Analysis of Factors Associated with Log-Transformed Renin and Aldosterone Levels at Day 5.

Variable	Log-Transformed Renin		Log-Transformed Aldosterone	
	β	*p*-Value	β	*p*-Value
Gestational age (weeks)	−0.06	0.24	−0.02	0.55
Multiple pregnancy	−0.06	0.65	−0.07	0.40
Cesarean section	0.11	0.17	−0.01	0.90
Use of antenatal steroids	−0.06	0.82	0.02	0.90
Use of tocolytic agents	−0.18	0.07	−0.08	0.22
Use of inotropic agents	−0.17	0.26	−0.01	0.91
Use of diuretics	−0.12	0.27	−0.23	<0.001
SGA	−0.17	0.19	−0.04	0.59
Weight change from birth (%)	−0.23	0.21	0.01	0.69
Nutrition (1, formula feeding, vs. 0, no enteral feeding)	−0.39	0.52	−0.27	0.50
Nutrition (2, mixed feeding, vs. 1, formula feeding)	−0.20	0.46	−0.18	0.29
Nutrition (3, breast milk, vs. 2, mixed feeding)	0.01	0.98	0.20	0.44

Data are multivariate linear regression coefficients (β) with *p*-values. The results were log-transformed. Nutrition was treated as an ordinal variable with four categories: 0 = no enteral feeding, 1 = formula feeding, 2 = mixed feeding, and 3 = breast milk. The significance threshold was set at *p* < 0.01.

## Data Availability

The original contributions of this study are included in this article. Further inquiries can be directed to the corresponding authors.

## Supplementary Materials

The following supporting information can be downloaded at: https://www.mdpi.com/article/10.3390/biomedicines14010064/s1, Figure S1: Relationship between day-0 aldosterone concentrations and birthweight; Table S1: Multivariable Linear Regression Analysis of Factors Associated with Aldosterone-to-renin ratio day 0; Table S2: Multivariable Linear Regression Analysis of Factors Associated with Aldosterone-to-renin ratio day 5.

## Abbreviations

The following abbreviations are used in this manuscript:

RAAS	Renin–angiotensin–aldosterone system
ACE	angiotensin-converting enzyme
AT1	angiotensin II type 1
PRA	plasma renin activity
PROM	premature rupture of membranes
HDP	Hypertensive disorders of pregnancy
SGA	Small for gestational age
CLEIA	chemiluminescent enzyme immunoassay
