# Supplementary material for: Postnatal Changes of Renin and Aldosterone in Term and Preterm Infants from Birth to Day 5
Source: Biomedicines. 2025 Dec 27;14(1):64. doi: 10.3390/biomedicines14010064 (PMC12837866; doi:10.3390/biomedicines14010064)
Supplement: Supplementary file 1 [file biomedicines-14-00064-s001.zip › biomedicines-3957985-supplementary.pdf]

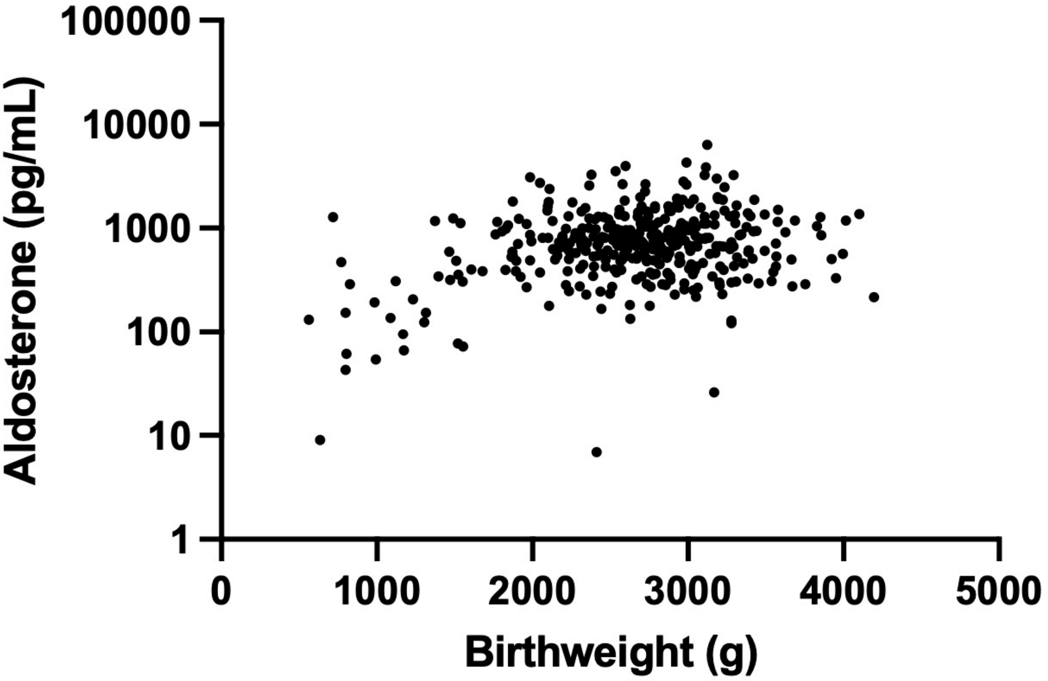

Supplementary Figure S1. Relationship between day-0 aldosterone concentrations and birthweight.

A scatter plot showing serum aldosterone concentrations measured at birth (day 0) plotted against birthweight in 374 infants. Aldosterone values demonstrated a distributional pattern similar to that observed with gestational age ($r = 0.145$, $p < 0.01$), suggesting that both overall body size and maturational factors may contribute to hormonal variability at birth.

Supplementary Table S1. Multivariable Linear Regression Analysis of Factors Associated With Aldosterone-to-renin ratio day 0

**Variable**	Aldosterone-to-renin ratio	
	β	p-value
Gestational age (weeks)	0.50	0.10
Multiple pregnancy	-5.10	<0.0001
Cesarean section	-1.02	0.17
Use of antenatal steroids	0.20	0.90
Use of tocolytic agents	-1.06	0.25
SGA	0.52	0.66

Data are multivariable linear regression coefficients (β) with p-values. The results were log-transformed. The significance threshold was set at $p<0.01$

Supplementary Table S2. Multivariable Linear Regression Analysis of Factors Associated With Aldosterone-to-renin ratio day 5

**Variable**	Aldosterone-to-renin ratio	
	β	p-value
Gestational age (weeks)	2.41	0.54
Multiple pregnancy	2.16	0.84
Cesarean section	-6.59	0.28
Use of antenatal steroids	-0.68	0.97
Use of tocolytic agents	8.53	0.26
Use of inotropic agents	8.05	0.50
Use of diuretics	0.66	0.94
SGA	7.08	0.49
Weight change from birth (%)	1.05	0.55
Nutrition (1, formula feeding, vs. 0, no enteral feeding)	-25.97	0.59
Nutrition (2, mixed feeding, vs. 1, formula feeding)	4.95	0.82
Nutrition (3, breast milk, vs. 2, mixed feeding)	-1.27	0.97

Data are multivariable linear regression coefficients (β) with p-values. Nutrition was treated as an ordinal variable with four categories: 0 = no enteral feeding, 1 = formula feeding, 2 = mixed feeding, and 3 = breast milk. The results were log-transformed. The significance threshold was set at $p<0.01$
